# A rare complication of an Arteriovenous shunt after bimaxillary surgery

**DOI:** 10.1016/j.jpra.2021.02.009

**Published:** 2021-03-11

**Authors:** A.R. Bouter, P.A. van Twisk, P.J. van Doormaal, B.J. Emmer, M.J. Koudstaal

**Affiliations:** a Department of Oral and Maxillofacial Surgery, Erasmus MC, University Medical Center Rotterdam, Netherlands; b Department of Radiology and Nuclear Medicine, Erasmus MC, University Medical Center Rotterdam, Netherlands; c Department of Radiology and Nuclear Medicine, Amsterdam University Medical Center, Netherlands

**Keywords:** Bimaxillary surgery, Vascular complications, Arteriovenous fistula, Embolization

## Abstract

The most common complications following bimaxillary surgery are inferior alveolar nerve damage, hemorrhage, and relapse. Severe complications are rare, but few cases of vascular arteriovenous malformation, cavernous sinus thrombosis, formation of an aneurysm or arteriovenous shunting are reported in literature. We present a case of a 20-year-old male patient who developed a right sided tinnitus and visible pulsations close to the mandibular angle on the right side after bimaxillary surgery. CT-angiography and subsequent digital subtraction angiography (DSA) six months after surgery showed an arteriovenous fistula (AVF) from the external carotid artery to the external jugular vein. The AVF was treated by endovascular coil embolization. At six months after intervention there were no residual complaints. We discuss the possible etiology and trauma mechanisms that might have caused this pathology and present recommendations to avoid this type of complications.

## Introduction

Bimaxillary surgery is a fairly common procedure globally and is performed regularly in various hospitals. The indications range from correcting dental malocclusion, obstructive sleep apnea as well as restoring the facial contour. Severe complications are rare. The most common complications reported are inferior alveolar nerve damage, wound dehiscence, intra –or postoperative hemorrhage, infected osteosynthesis material and TMJ dysfunction.^1^ We would like to present a rare complication of an arteriovenous fistula following bimaxillary surgery. For this case report, STROBE guidelines were followed.

## Report of a case

A 20-year old male patient was referred to us for a second opinion by his orthodontist, 6 months after having bimaxillary surgery which was performed in another hospital. The indication for bimaxillary surgery had been a skeletal class III malocclusion with frontal open bite. A le Fort 1 osteotomy with 3 mm dorsal impaction and 4 mm advancement and a BSSO setback of 4 mm was performed according to the notes.

His-main complaint when visiting our department was a tinnitus on the right, which had started shortly following the bimaxillary surgery. Clinical examination showed a pulsating mass just lateral of the mandibular angle on the right side. There was a class I occlusion without TMJ complaints or restricted mouth opening. Impaired sensation of the mental nerve on both sides was present.

The bimaxillary surgery took approximately 3 h and a total blood loss of 2850 ml was noted. No further anomalies were reported. After surgery a prolonged stay on the post-operative ward of three hours in total was indicated. This was due to laryngospasm which was attributed to blood on the vocal cords.

### Diagnostics and treatment

**Fig. 1:**
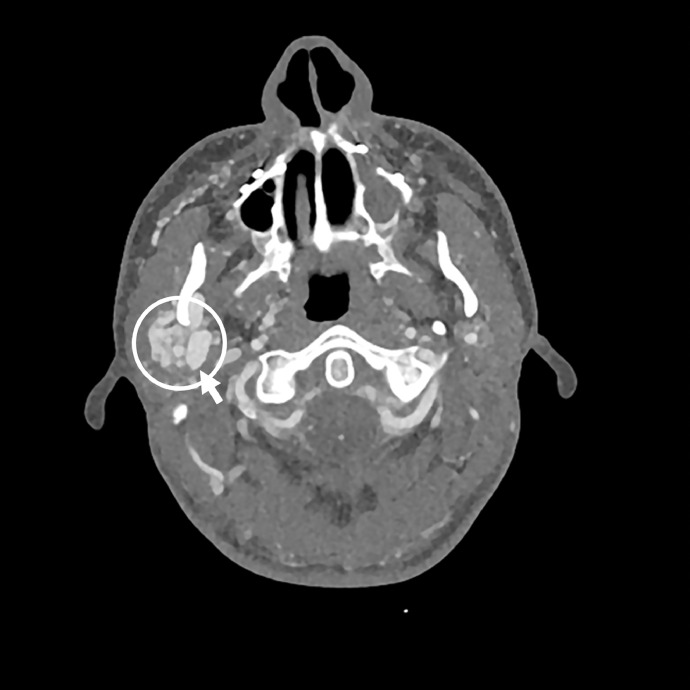
Axial slice of the performed CTA showing abnormal dilated vessels in the right parotid space (circle). There is early filling of the right external jugular vein compared to the left side, suggestive of an arteriovenous communication.

**Fig. 2:**
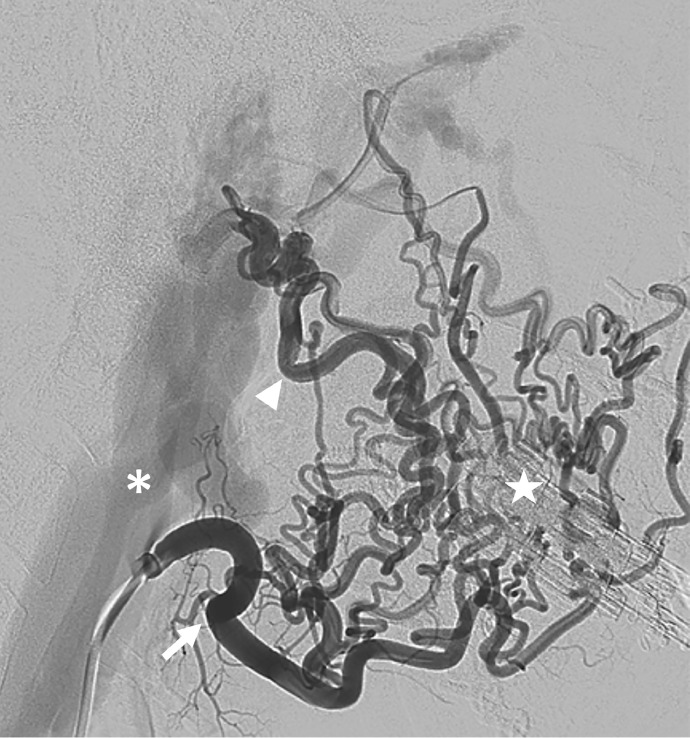
Lateral digital subtraction angiography (DSA).Contrast injection in the right facial artery showing multiple hypertrophic branches of the facial artery (arrow) and of the inferior alveolar artery (arrowhead) via the internal maxillary artery. Note that all branches are directed towards one anatomical location (star), with signs of an obvious nidus. Early filling of the dilated external jugular vein (asterisk) and the common jugular vein, suggestive of an arteriovenous fistula (AVF).

Because of the tinnitus and pulsating mass in his neck, vascular pathology was suspected, and a CTA was performed showing hypertrophied arteries and dilated draining veins with marked arterial enhancement (Fig. 1). The patient was referred to an interventional neuroradiologist and an additional digital subtraction angiography (DSA) confirmed the presence of an arteriovenous fistula on the right external jugular vein, fed by multiple hypertrophied branches arising from the right facial and internal maxillary artery (Fig. 2). The patient was scheduled to undergo embolization under general anesthesia. The branches of the external carotid artery were selectively catheterized to get an overview of the fistula. Through a trans-venous approach, the external jugular vein as the primary draining vein was embolized using a combination of bare platinum and fibered coils.

### Follow-up

No complications occurred during the procedure. The right sided tinnitus disappeared immediately following the embolization. Three days after embolization the patient developed pain in the mandibular angle region. Echography showed a thrombus in the external jugular vein in accordance with the recent treatment and no sign of bleeding or residual flow from the occluded vessels. The pain was successfully managed using analgesics.

**Fig. 3:**
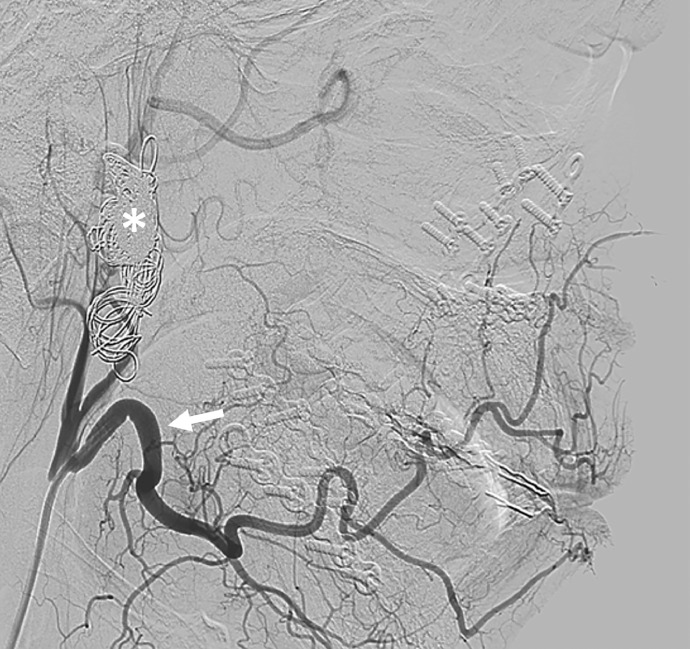
Follow-up angiography 6 months after embolization with coils (asterisk) showing no signs of early venous filling. Normalization of the previously facial artery (arrow) and inferior alveolar artery (not visible here). Absence of early venous filling and disappearance of previously hypertrophied feeding vessels are compatible with absence of a residual fistula.

**Fig. 4:**
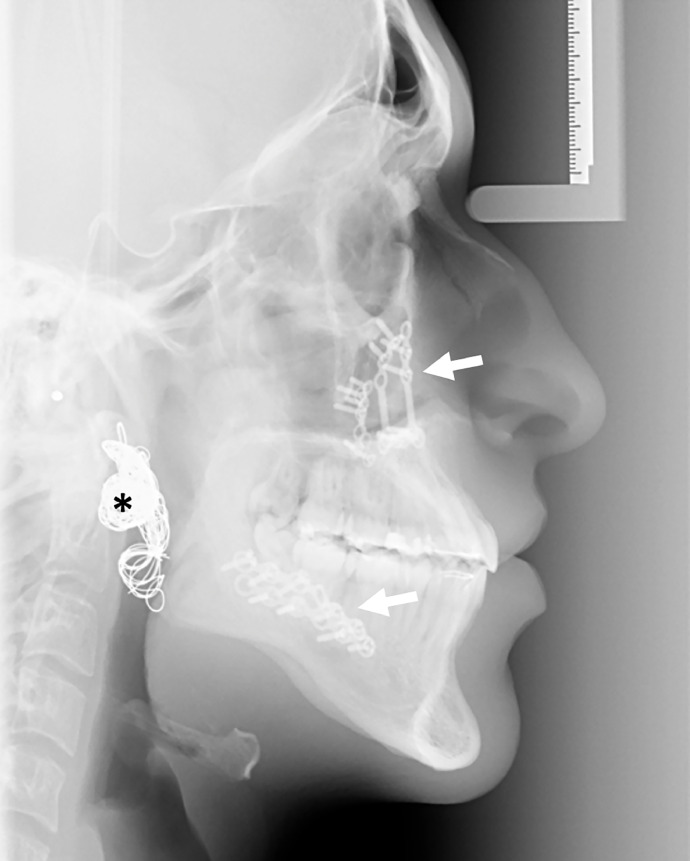
Lateral cephalogram 6 months after embolization showing a class I occlusion with osteosynthesis material (arrows) and coils (asterisk) in the right parotid space.

After 6 months of follow-up there were no complaints. Clinical examination showed the preexistent impaired sensation of the lower lip. To confirm the complete occlusion of the fistula a control DSA was performed, showing no sign of a residual fistula. The lateral cephalogram showed a class I occlusion with osteosynthesis materials and coils in situ (Figs. 3 and 4).

## Discussion

Bimaxillary surgery is usually associated with minor complications. Infrequently but severe complications like blindness following le Fort I osteotomy are however reported.^2^ Vascular complications like cavernous sinus thrombosis, formation of an aneurysm or arteriovenous fistula like in our patient are rare.^3^ During the le Fort I osteotomy the descending palatine artery and the internal maxillary artery are at risk due to its proximity to the pterygomaxillary junction in the pterygopalatine fossa.^4^ During separation of the pterygoid plates using an osteotome, laceration of the internal maxillary artery can occur thus causing vascular complications like formation of a (pseudo) aneurysm.^5^^,^^6^ Also formation of direct fistulae of the internal carotid artery and internal jugular vein or sinus cavernous are described.^7^

During the bilateral sagittal split osteotomy laceration of the facial artery can occur when the lower border is cut without appropriate protection. Pseudo aneurysm and arteriovenous fistulae of the facial artery as well as the external carotid artery are reported.^8^^,^^9^ It is believed that arteriovenous shunting is caused by incomplete laceration of an artery with simultaneously laceration of a vein.^3^ The subsequent formation of a hematoma is subjected to endothelial proliferation and formation of endothelial lined channels between the artery and the vein. After this, blood is shunted from the arterial high-pressure system to the venous low-pressure system. Pulsatile tinnitus like in our patient can occur due to the preferred flow into the AV-fistula and resonating of the shunt wall.^10^

In our patient the arteriovenous fistula had feeding vessels from the facial and maxillary artery with involvement of the external jugular vein. Given the timing of onset of complaints and the performed surgery in which different vascular structures are at risk, we believe that an iatrogenic origin of the arteriovenous fistula with laceration of one of the above-mentioned vessels is most likely. Although it seems unlikely, given the patient had no complaints prior to the operation, pre-existent presence of a vascular anomaly or arteriovenous malformation can however not be excluded.

This case shows the importance of sufficient peroperative soft tissue protection, especially during exposure of the mandibular ramus and cut of the lower border for the bilateral sagittal split osteotomy and during the posterior osteotomy and separation of pterygoid plates for the le Fort I osteotomy. This can be established through correct use of surgical retractors and awareness of the anatomical structures that are at risk. Furthermore, it is important to be aware of the possible occurrence of vascular complications like arteriovenous fistulae as described in this report.

## Conclusion

Arteriovenous shunting following orthognathic surgery is a rare but serious complication. During le fort I osteotomy the internal maxillary artery and internal carotid artery and during sagittal split osteotomy and the facial artery are mainly at risk. In this report we presented a case of an arteriovenous fistula of the internal maxillary artery and the external jugular vein, most likely due to direct arterial damage during bimaxillary surgery. The fistula was successfully treated through embolization. To prevent severe vascular complications like this, sufficient soft tissue protection is of importance.

## Declaration of Competing Interest

The authors have no conflict of interest to declare.

## References

[1] Jedrzejewski M., Smektala T., Sporniak-Tutak K. (2015). Preoperative, intraoperative, and postoperative complications in orthognathic surgery: a systematic review. Clin Oral Investig.

[2] Cruz A.A., dos Santos A.C. (2006). Blindness after Le Fort I osteotomy: a possible complication associated with pterygomaxillary separation. J Craniomaxillofac Surg.

[3] Lanigan D.T., Hey J.H., West R.A. (1991). Major vascular complications of orthognathic surgery: false aneurysms and arteriovenous fistulas following orthognathic surgery. J Oral Maxillofac Surg.

[4] Smith I.M., Anderson P.J., Wilks M.J., David D.J. (2008). Traumatic arteriovenous malformation following maxillary Le Fort I osteotomy. Cleft Palate Craniofac J.

[5] Hacein-Bey L., Blazun J.M., Jackson R.F. (2013). Carotid artery pseudoaneurysm after orthognathic surgery causing lower cranial nerve palsies: endovascular repair. J Oral Maxillofac Surg.

[6] Silva A.C., O'Ryan F., Beckley M.L. (2007). Pseudoaneurysm of a branch of the maxillary artery following mandibular sagittal split ramus osteotomy: case report and review of the literature. J Oral Maxillofac Surg.

[7] Osborne P.R., Sverzut T.F.V., Trivellato A.E. (2017). Carotid jugular fistula after Le Fort I osteotomy. Int J Oral Maxillofac Surg.

[8] Jo H.W., Kim Y.S., Kang D.H. (2013). Pseudoaneurysm of the facial artery occurred after mandibular sagittal split ramus osteotomy. Oral Maxillofac Surg.

[9] Pappa H., Richardson D., Niven S. (2008). False aneurysm of the facial artery as complication of sagittal split osteotomy. J Craniomaxillofac Surg.

[10] Albernaz V.S., Tomsick T.A. (1995). Embolization of arteriovenous fistulae of the maxillary artery after Le Fort I osteotomy: a report of two cases. J Oral Maxillofac Surg.

